# Trans-cranial focused ultrasound without hair shaving: feasibility study in an ex vivo cadaver model

**DOI:** 10.1186/2050-5736-1-24

**Published:** 2014-01-02

**Authors:** Matthew DC Eames, Arik Hananel, John W Snell, Neal F Kassell, Jean-Francois Aubry

**Affiliations:** 1 Focused Ultrasound Foundation, Charlottesville, VA 22903, USA; 2 Department of Radiation Oncology, University of Virginia, Charlottesville, VA 22908, USA; 3 Department of Neurosurgery, University of Virginia, Charlottesville, VA 22908, USA; 4 Institut Langevin Ondes et Images, ESPCI ParisTech, CNRS UMR 7587, Inserm U979, Paris 75238, France

**Keywords:** tcMRgFUS, Brain, Hair, MR thermometry

## Abstract

In preparing a patient for a trans-cranial magnetic resonance (MR)-guided focused ultrasound procedure, current practice is to shave the patient’s head on treatment day. Here we present an initial attempt to evaluate the feasibility of trans-cranial focused ultrasound in an unshaved, *ex vivo* human head model. A human skull filled with tissue-mimicking phantom and covered with a wig made of human hair was sonicated using 220- and 710-kHz head transducers to evaluate the feasibility of acoustic energy transfer. Heating at the focal point was measured by MR proton resonance shift thermometry. Results showed that the hair had a negligible effect on focal spot thermal rise at 220 kHz and a 17% drop in temperature elevation when using 710 kHz.

## Introduction

Focused ultrasound (FUS), or high-intensity focused ultrasound (HIFU), involves depositing ultrasonic energy into a target volume, where the area of acoustic emission is significantly greater than the focal area. This focusing effect allows generation of a high level of acoustic intensity at the target volume, thereby triggering and amplifying a variety of bio-effects ranging from thermal to mechanical. Currently, the most common usage of HIFU in clinical practice is for noninvasive thermal ablation with close to 100,000 patients treated worldwide, mainly for the indications of prostate cancer [1,2], liver cancer [3-5], breast cancer [6,7], and symptomatic uterine fibroids [8-10].

Treatments are performed under image guidance, either by magnetic resonance imaging (MRI; MRgFUS, MR-guided FUS) [11,12] or by ultrasonic imaging (USgFUS, US-guided FUS) [13,14].

HIFU, being a noninvasive, accurate, radiation-free thermal ablation tool, has long been viewed as an ideal treatment tool for various brain indications [15-17]. However, the usage of HIFU for the brain is hampered by the defocusing of the ultrasonic beam by the patient skull [18,19]. The development of aberration correction techniques [20-22] has led to a significant increase in clinical research involving trans-cranial MRgFUS (tcMRgFUS), mainly as a functional neurosurgery tool for treatment of essential tremor [23] and neuropathic pain [24] with more than 80 patients treated worldwide to date.

Current practice requires shaving the patient’s head on tcMRgFUS treatment day. This practice, although noninvasive and scientifically and clinically sound, is often a concern to patients. In addition, it presents a potential limitation if and when there will be a need for a repeated tcMRgFUS in the cases of BBB opening [25-27] that should be synced to chemotherapy administration or when ‘time to treat’ may be limited, as could be the case in stroke clot lysis [28,29]. Earlier work by Raymond and Hynynen [30] has shown in a lab model that insertion loss due to strands of hair aligned perpendicular to beam propagation is frequency dependent and that it is less than 20% for frequencies less than 1.7 MHz.

Here we present an initial attempt to evaluate the feasibility of tcMRgFUS in a close to full clinical model using an *ex vivo* cadaver skull with and without a wig made of human hair.

## Methods

**Figure 1:**
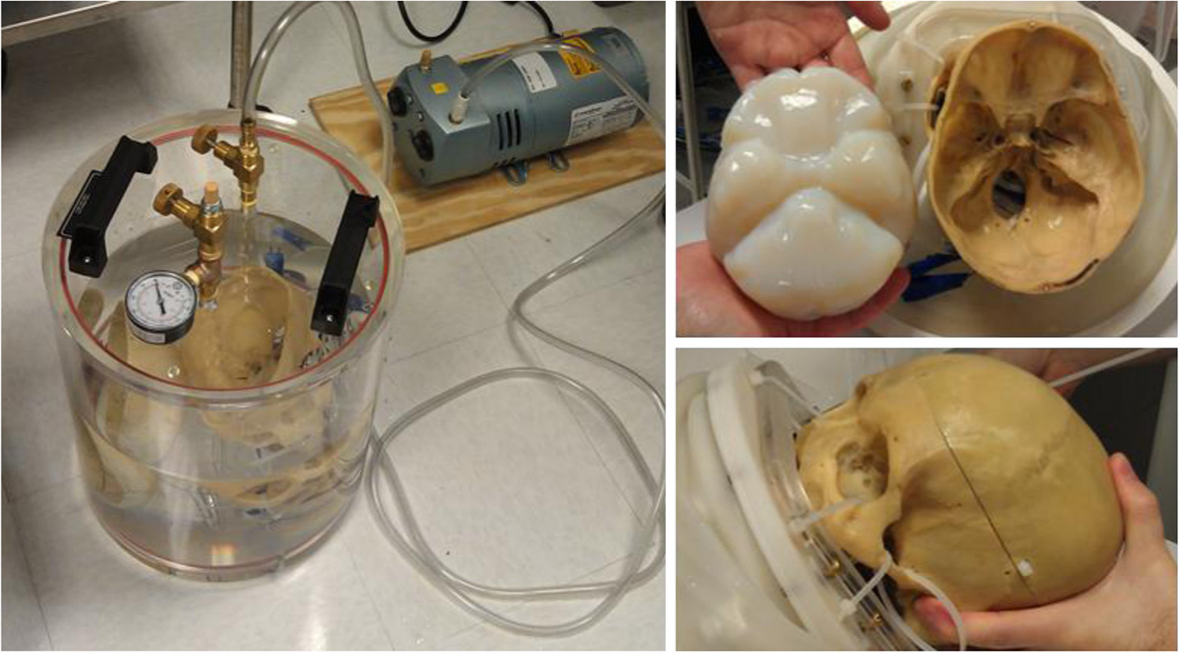
Degassing of the cadaver skull, filling with tissue-mimicking phantom, and setting for placement inside the ExAblate-Neuro.

A human cadaveric skull was recovered from a cadaver obtained through the Virginia Department of Health State Anatomical Program. The skull was defleshed and cleaned and then stored for 12 months in air at room temperature before starting the current experiment. To build a model as close as possible to a patient’s head, in terms of size and acoustic properties, the skull was filled with tissue-mimicking hydrogel (ATS Laboratories, Bridgeport, CT, USA) (Figure 1). Acoustic properties of the gel provided by the manufacturer were as follows: speed of sound (1,540 m/s), absorption coefficient (0.5 dB/cm/MHz). The hydrogel was melted in an 800-W microwave (Oster model OM0701A8B, Foshan, Guangdong, China) at 50% power for 12 min. The temperature of the hydrogel right after melting in the microwave was 51°C. The inside of the upper and lower halves of the skull were lined with plastic film to create a watertight mold into which the gel was poured and cooled to approximately 5°C for at least 8 h to solidify.

The human *ex vivo* skull was degassed in water with less than 2 ppm oxygen for 20 min in a vacuum chamber (Acrylic Round Vacuum Chamber, Abbess, Holliston, MA, USA) at 230 mmHg. Once degassed, the upper and lower gel molds were placed inside the upper and lower parts of the skull while still submerged in water. The two halves of the skull were connected together while submerged in water. For the cases in which a wig was used, it was submerged in the degassed water, gently stirred to remove trapped air bubbles and then placed on the skull. The skull assembly was then mounted in the ExAblate-Neuro (InSightec, Tirat Hakarmel, Israel) ultrasound transducer, which was promptly filled with water to minimize the presence of air in the experimental setup. This process was repeated with two ExAblate-Neuro transducers, each with a different operating frequency (220 and 710 kHz).

**Figure 2:**
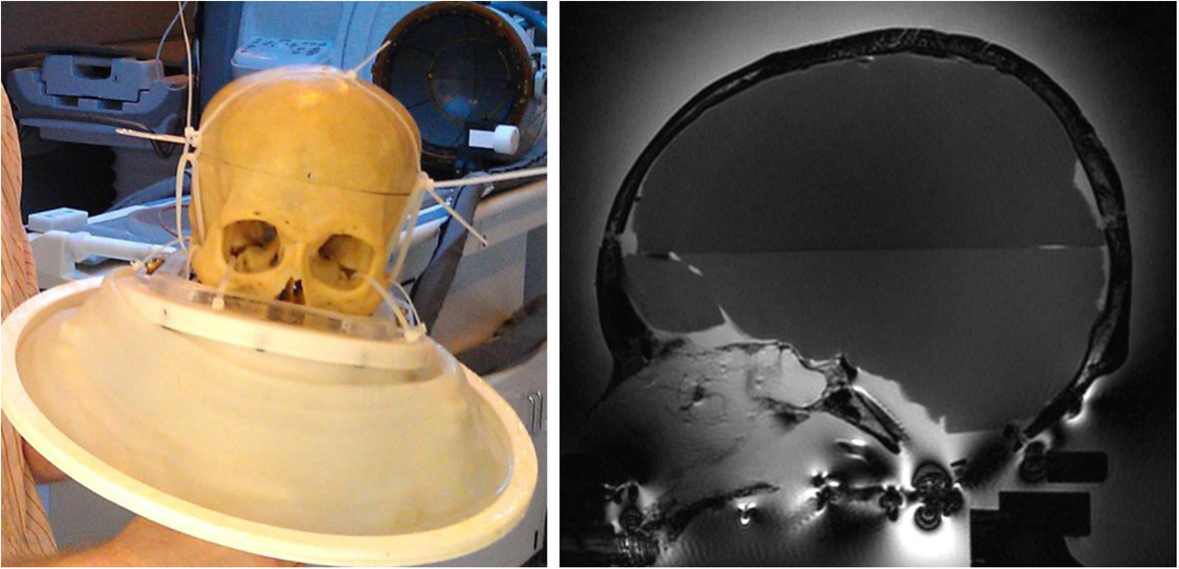
**Bare skull strapped to holder (*left*) and as seen on MR T2w sagittal image (*right*).**

**Figure 3:**
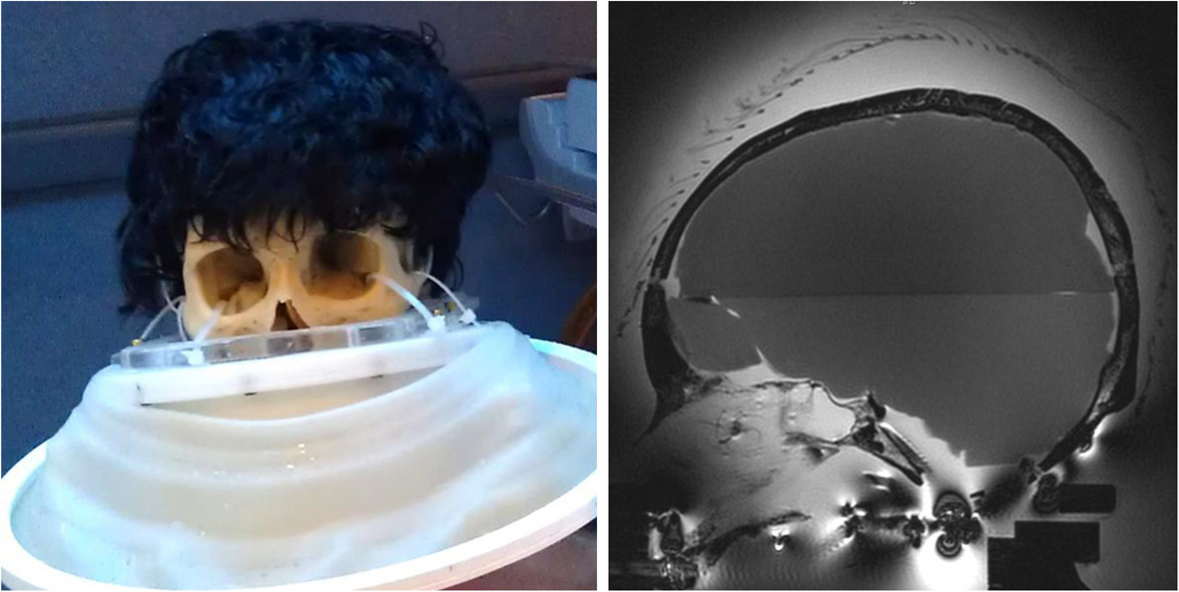
**Skull with wig strapped to holder (*left*) and as seen on MR T2w sagittal image (*right*).**

**Figure 4:**
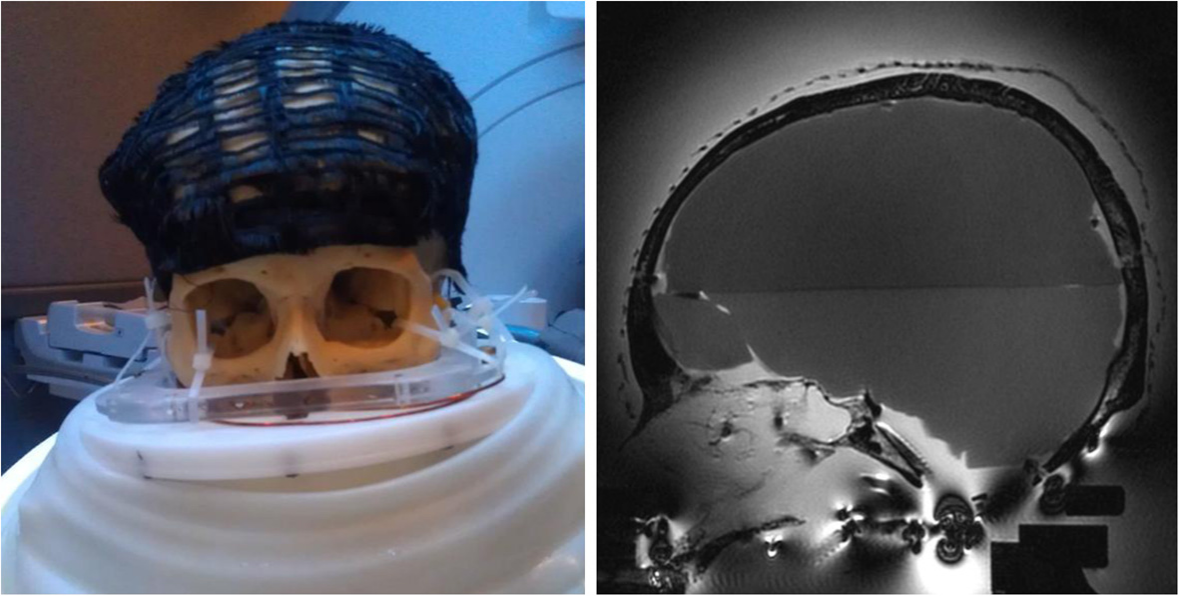
**Skull with wig cap strapped to holder (*left*) and as seen on MR T2w sagittal image (*right*).**

The hair is attached to the wig by the manufacturer with a dedicated cap. In order to investigate the relative influence of the hair and the cap, three setups were tested for each transducer: the bare skull (Figure 2), the same skull covered with a human hair wig (H-222, color 1, by Vivica Fox, Vivica A. Fox Hair Collection, Conshohocken, PA, USA; Figure 3), and the skull covered with only the cap part of the wig (used as baseline for the wig setup; Figure 4) after cutting the hair.

For each of the above setups, multiple 10-s-duration sonications were performed with increasing power. Power range varied between 120 and 420 W (120, 180, 240, 300, 360, and 420 W) with 10-s duration and duty cycle of 100%.

During energy deposition, gel thermal rise in the focal point and near the skull was evaluated using the proton resonance frequency (PRF) shift method of MR temperature mapping. The MR system was a 3T discovery (GE, Milwaukee, WI, USA). The MR thermometry scan parameters used were as follows: TR/TE 27.6/12.8 ms, flip angle 30°, bandwidth 5.68 kHz, FOV 28 cm, slice thickness 3 mm, matrix 256 × 128, scan time 3 s. A temperature sensitivity of -0.009 ppm/°C was used [31]. Three independent measurements were performed for each power and each configuration. Thermal rise at the focus as a function of power was fitted with a constrained linear least squares method (MATLAB, MathWorks, Natick, MA, USA).

## Results

**Figure 5:**
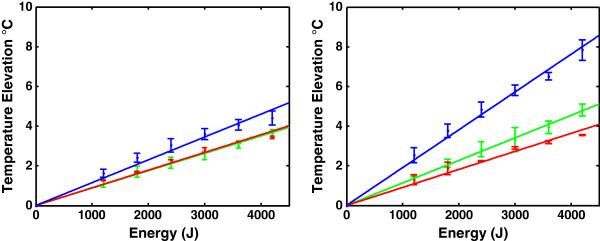
**Temperature elevation as a function of energy.** Measured heating using 220 kHz (*left*) and 710 kHz (*right*) in bare (*blue*), cap (*green*), and wig (*red*) skull setups. Standard deviation is displayed as *error bars*.

Temperature elevation is plotted as a function of energy in Figure 5 for the 220- (left) and 710-kHz (right) setups for each configuration: the entire wig (wig and cap), the cap only, and the skull alone (no wig and no cap). Results of the corresponding constrained linear least squares fit are summarized in Table 1.

**Table 1:** Slope of the linear least squares fit for each configuration

	**System (°C/J)**	
	**220 kHz**	**710 kHz**
No wig and no cap	1.1 × 10^-3^	1.9 × 10^-3^
Cap alone	0.88 × 10^-3^	1.1 × 10^-3^
Cap and wig	0.89 × 10^-3^	0.91 × 10^-3^

**Figure 6:**
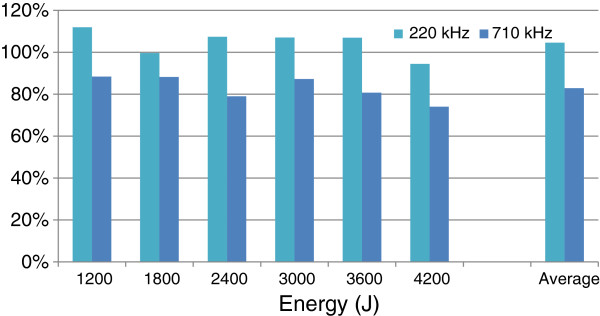
Wig to cap thermal rise ratio using 220 and 710 kHz.

Temperature elevation with and without the wig showed a 19% decrease at 220 kHz and 53% at 710 kHz. One can notice that most of the attenuation is in fact linked to the presence of the cap: there is no noticeable difference in temperature rise between the cap and the cap-and-wig setup at 220 kHz, and there is a limited 17% reduction at 710 kHz. The frequency-dependent impact on acoustic transmission can be better visualized by comparing the hair to cap ratio in both frequencies (Figure 6).

## Discussion

The results show that the hair induces a minimal additional loss compared to the skull itself. The effect of the hair alone, after taking into account the effect of the cap, corresponds to a 17% decrease in temperature elevation at the focus at 710 kHz and no noticeable change at 220 kHz. Temperature elevation at the focus, being proportional to the square of the focal pressure, corresponds to a 4% decrease in the pressure at 710 kHz and no significant decrease at 220 kHz. These numbers need to be discussed in the light of the energy loss associated with the errors induced by imperfect phase aberration correction. Current noninvasive aberration correction techniques are not perfect, and this leads to a significant drop in the final focal temperature rise as compared to the best correction possible, achievable with an implanted hydrophone. As a matter of fact, this drop is on the order of that induced by the presence of the wig and cap at 710 kHz. CT-based corrections have been reported to restore 70% of the pressure at the focus at 660 kHz [19] and 85% at 1 MHz [30,31], as compared to hydrophone-based gold standard corrections. Based on the squared relationship between pressure and temperature, the aberration correction process yields a focal temperature rise that is 49% of that obtained with gold standard correction at 660 kHz, corresponding to a 51% drop in temperature elevation at the focus.

This is consistent with the fact that the human hair is expected to have minimal scattering effect because of its mean diameter. The human hair diameter is typically in the range between 30 and 110 μm [32] and is thus negligible compared to the ultrasonic wavelength (6.8 mm at 220 kHz and 2.1 mm at 710 kHz).

Raymond and Hynynen reported similar results on acoustic transmission through homemade hair-mat phantoms made by placing aligned human hairs lengthwise between two acrylic supports [30], with a hair density varying from 294 to 521 hairs/cm. Within the range of frequencies studied here, they reported transmission loss lower than 20% with the hair phantom oriented perpendicular to beam propagation, whatever the hair density. Our results suggest that these findings remain valid in a more realistic geometry of the hair and scalp. One has to mention that the implementation of the natural hair on the commercial wig was enabled by the manufacturer with the use of a cap, the substructure to which the hair was attached (Figure 4). Such a cap is affecting the transmission of the beam, as can be seen in Table 1: 20% relative drop in temperature elevation with the 220-kHz array and 42% with the 710-kHz array. Patients undergoing tcMRgFUS treatment are currently shaved and do not have such a cap. Most of the comments in the discussion use the cap as a reference to investigate the influence of the hair alone.

One can notice that four of the six wig-to-cap ratios are greater than 100% for the 220-kHz array (Figure 6). The presence of the hair is nevertheless expected to decrease the transmission of ultrasound and thus decreases the temperature at the focus. As a matter of fact, all the corresponding measurements are close to 100%: the average absolute difference between the temperature elevations obtained with and without the wig for the 220-kHz array is 0.2°C, which is on the order of the precision of the MR temperature measurement.

The results show that the efficiency of the treatment is unlikely to be compromised by the presence of the hair. Nevertheless, even though only a fraction of the energy is lost when sonicating through the hair, part of this loss is likely to be absorbed by the hair and could potentially lead to skin burns. The corresponding temperature elevation could not be measured here due to the presence of water only between the hair and the skull. In order to further investigate the temperature elevation in the hair and close to the hair, a more detailed model of a human head would have to be developed, including not only brain tissue-mimicking phantom, wig, and skull but also skin or embedding the hair in a gel. MR temperature monitoring during treatment is currently limited to one sagittal or coronal plane [23,24]. Current developments include full 3D MR thermometry of the whole brain volume [33]. Such methods could be extended in the future to monitor the temperature of the skin itself in the case of unshaved treatment.

One last difficulty for trans-hair treatment is that air bubbles can be trapped in the hair. Air bubbles are known to not only block ultrasound but also absorb ultrasonic energy. In this study, the wig was gently stirred by hand while submerged in the degassed water to remove trapped air bubbles. Such a hand stirring is possible to achieve in the current clinical setup, and circulation of degassed water is contributing to remove air bubbles once everything was in place.

## Conclusions

Based on these very initial results and assuming that our full-scale model of cadaver skull and human hair wig setup is sufficiently similar to the clinical scenario, it should be possible to deliver trans-cranial focused ultrasound brain thermal ablation using either 220- or 710-kHz central frequency without shaving the hair. Further studies are needed to make sure that it does not result in significant thermal rise on the hair surface and on the skin.

## Competing interests

Matthew DC Eames, John W Snell, and Jean-Francois Aubry declare that they have no competing interests. Arik Hananel and Neal F Kassell have shares in InSightec, the company which manufactures the ExAblate device.

## Authors’ contributions

MDCE, JWS, and AH performed the experiments. MDCE, JWS, AH, and J-FA conducted the data analysis and participated in writing the paper. NFK directed and supported the research. All authors read and approved the final manuscript.
